# Effect of cryotherapy on arteriovenous fistula puncture-related pain in hemodialysis patients

**DOI:** 10.4103/0971-4065.45290

**Published:** 2008-10

**Authors:** Sabitha P. B., D. C. Khakha, S. Mahajan, S. Gupta, M. Agarwal, S. L. Yadav

**Affiliations:** College of Nursing, All India Institute of Medical Sciences, New Delhi - 110 029, India; 1 Department of Nephrology, All India Institute of Medical Sciences, New Delhi - 110 029, India; 2 Department of Physical Medicine and Rehabilitation, All India Institute of Medical Sciences, New Delhi - 110 029, India

**Keywords:** Cryotherapy, hemodialysis, puncture pain, subjective and objective assessment of pain

## Abstract

Pain during areteriovenous fistula (AVF) cannulation remains a common problem in hemodialysis (HD) patients. This study was undertaken to assess the effect of cryotherapy on pain due to arteriovenous fistula puncture in hemodialysis patients. A convenience sample of 60 patients (30 each in experimental and control groups) who were undergoing hemodialysis by using AVF, was assessed in a randomized control trial. Hemodialysis patients who met the inclusion criteria, were randomly assigned to experimental and control groups using a randomization table. Objective and subjective pain scoring was done on two consecutive days of HD treatment (with cryotherapy for the experimental and without cryotherapy for the control group). The tools used were a questionnaire examining demographic and clinical characteristics, an observation checklist for assessing objective pain behavior, and a numerical rating scale for subjective pain assessment. Descriptive statistics were used as deemed appropriate. Chi square, two-sample and paired t-tests, the Mann Whitney test, Wilcoxon's signed rank test, the Kruskal Wallis test, and Spearman's and Pearson's correlations were used for inferential statistics. We found that the objective and subjective pain scores were found to be significantly (*P* = 0.001) reduced within the experimental group with the application of cryotherapy. This study highlights the need for adopting alternative therapies such as cryotherapy for effective pain management in hospital settings.

## Introduction

Chronic renal failure is a devastating medical, social, and economic problem for both patients and their families in India. Most CKD patients reporting to tertiary care centers in India are in the final stage where renal replacement therapy (RRT) is the only option at that stage.^1^ Hemodialysis (HD) is the most frequently used RRT with the arteriovenous fistula (AVF) being the gold standard for vascular access in HD patients.^2^

Pain inflicted by the insertion of large cannulae into the AVF is a significant cause of concern for both children and adults on regular HD. Although AVF puncturing causes pain,^3^ local anesthesia is not frequently used due to concerns of vasoconstriction, burning sensation, scarring, and infection.^4^ On an average, a patient on maintenance hemodialysis undergoes ten AV fistula punctures a month and would continue to do so throughout their lifetime or until a successful renal transplant. His or her comfort with the procedure is therefore of utmost importance for long-term compliance with the treatment.

Research evidence shows that cutaneous stimulation^5^ is an independent nursing intervention that is advocated to minimize pain in patients.^6^,^7^ The effect of cutaneous stimulation is best explained by the gate control theory.^8^ Cutaneous stimulation modalities can be clubbed with accupressure to increase its effectiveness in pain management. The large intestine meridian is an acupressure point located on the back side of the hand between the thumb and the first finger. Its dominant uses are to relieve pain in the shoulder and arm, rigidity of the neck, scapula, and eye diseases, and to treat constipation or other bowel disorders.^9^ Studies have also thrown light on the fact that cryotherapy is equally effective in alleviating pain as a cutaneous stimulation technique.^10^–^12^ This study was therefore undertaken to look at the effect of cryotherapy on pain due to arteriovenous fistula puncture in hemodialysis patients, and to correlate the perceived pain with selected variables such as age, sex, duration of AV fistula use, and the educational status of the subjects.

## Materials and Methods

Patients undergoing hemodialysis via an AV fistula in the Dialysis unit of a tertiary care hospital, and who were older than 16 years of age, capable of giving adequate response to pain, and not having any other problem resulting in pain were enrolled for this randomized control trial. Patients having radiation injuries, peripheral vascular diseases, Raynauld's syndrome, connective tissue disorders, diabetic neuropathy, unconsciousness or disorientation, and requiring more than one attempt for fistula puncturing were excluded from this study. All the patients having an AVF in our unit and who fulfilled the inclusion criteria during the study period were approached. Sixty-eight patients were screened, of whom two refused consent, hence, 66 were randomly assigned to the experimental and control groups using a randomization table. Thus, 60 patients completed the study, of whom six were drop-outs (they had undergone multiple punctures or developed aneurysm). Thus, 30 patients each in the experimental and control groups completed the study.

The tools used for data collection were a questionnaire for collecting demographic and clinical data, an observation checklist for assessing objective pain behaviors, and a numerical rating scale for subjective pain assessment. Experts had established the content validity of the tools. The observation checklist was a five-point scale that rated objective pain behaviors^13^ into five subheadings: Facial expression, Body language, Verbalization, Biophysiological measures, and Interpersonal behavior. The reliability of the observation checklist was established by a test-retest method on 20 hemodialysis patients. The tool was found to be 72% reliable (r = 0.72) and the inter-rater reliability was found to be 89% (r = 0.89). The numerical rating scale, a standardized pain scale, has a well-established reliability of r = 0.78−0.93.^14^ Ethical clearance for conducting this study was obtained from the local Ethics Committee.

Hemodialysis patients who met the inclusion criteria were enrolled in the study and consent forms were signed before their participation in the study. Demographic data were collected by interviews using the questionnaire. Random assignment to the experimental and control groups was done based on a randomization table. Objective and subjective pain scoring was done on two consecutive days of hemodialysis treatment, without intervention in the control group and with cryotherapy on the second visit in the experimental group. The same group of staff members performed the punctures for both experimental and control group patients as per the unit schedule. Thus, the practical experience of staff members performing the punctures was comparable within the two groups.

### Intervention (cryotherapy)

Cold application was done with ice cubes wrapped in gloves on the web between the thumb and index finger of the hand not having the AV fistula (contralateral arm). The procedure was started ten minutes before venipuncture and continued throughout the puncturing procedure (approximately two minutes). The researcher herself performed the ice massage, while another staff member did the AVF cannulation (SPB).

### Statistical analysis

Data analysis was done using STATA; both descriptive and inferential statistics were used. Descriptive statistics used in the study were frequencies, percentage, mean, median, range, and standard deviation. Chi square, two-sample and paired t-tests, the Mann Whitney test, Wilcoxon's signed rank test, the Kruskal Wallis test, Spearman's and Pearsons correlations were used for inferential statistics as deemed appropriate.

## Results

The experimental and control groups were comparable with respect to demographic and treatment-related variables such as age (*P* = 0.87), the duration of HD (*P* = 0.15), the duration of AV fistula use (*P* = 0.06), sex (*P* = 1.00), educational qualification (*P* = 0.13), and occupation (*P* = 0.73) of the subjects [Table 1]. However, there were more patients with diabetes mellitus in the control group as compared to the experimental arm (43.3 *vs* 20%, *P* = 0.05).

**Table 1:** Demographic and treatment modality-related characteristics of HD patients

Variable	Experimental group (n = 30)	Control group (n = 30)	*P* value
Age (Median, years)	35 (17–66)	33.5 (18–69)	0.87
Duration on HD (Median, months)	6 (2–36)	6 (1–24)	0.15
Duration of AVF use (Median, months)	5 (0.25–23)	2.5 (0.25–18)	0.06
Male (%)	23 (76.7)	23 (76.7)	1.00
Graduates (%)	14 (46.7)	20 (66.7)	0.13
Hypertension (%)	20 (66.7)	18 (60.0)	0.59
Diabetes mellitus (%)	6 (20.0)	13 (43.3)	0.05
HCV-positive (%)	4 (13.3)	6 (20.0)	0.49
Coronary artery disease (%)	6 (20.0)	7 (23.3)	0.75

There was no association between the baseline (day 1 of HD) pain scores (objective and subjective) and the selected variables such as the duration of AV fistula use, educational qualification, and the age of the subjects. Females however, had significantly higher objective (4.5 ± 0.65 *vs* 3.5 ± 0.68, *P* = 0.01) and subjective (4.5 ± 1.6 *vs* 3.6 ± 1.8, *P* = 0.03) pain scores as compared to the male subjects.

The objective AV fistula puncture pain scores on days 1 and 2 of HD within the experimental group were found to be significantly reduced (*P* = 0.001) from an average of 3.8 on day 1 of HD (when the patient received routine care) to 0.7 on day 2 of HD (when cryotherapy was given). There was a significant reduction (*P* = 0.001) in the subjective AV fistula puncture pain scores (1−2.5) on day 2 of HD as compared to the scores (2−7) on day 1 of HD.

AV fistula puncture pain scores on days 1 and 2 of HD within the control group were found to be similar (*P* = 0.23) on two consecutive days of HD (when patient received only routine care). There also was no significant difference (*P* = 0.89) in subjective AV fistula puncture pain scores on day 2 of HD as compared to the scores on day 1 of HD [Table 2].

**Table 2:** Comparison of objective and subjective pain scores on days 1 and 2 of hemodialysis in two groups

Pain scores	Objective pain score Mean (SD)		Subjective pain score Median (Range)	
		
	Experimental	Control	Experimental	Control
Day 1 of HD (1.5–6.5) (Baseline)	3.8 (0.66)	3.6 (0.73)	4.5 (2–7)	3.2
Day 2 of HD (1.5–7)	0.7 (0.33)	3.5 (0.76)	2 (1.2–5)	3.5
*P* value	0.001	0.23	0.001	0.89

HD: Hemodialysis

## Discussion

Hemodialysis patients^3^,^4^ frequently report pain during AVF puncture and alleviation of this pain might improve their acceptance of the procedure and thus, their quality of life. This study is the first study of this kind in which the effect of cryotherapy was studied as a cutaneous simulation technique in the reduction of invasive pain such as that of AV fistula puncture. In the present study, the objective and subjective pain scores were found to be significantly (*P* = 0.001) reduced within the experimental group with the application of cryotherapy. Thus, cryotherapy was found to be an effective pain management technique in these patients. Studies using cutaneous stimulation to decrease acute pain have shown the benefit of these procedures. In 1980, Melzack *et al*.^11^ conducted a study in which patients suffering from acute dental pain were treated with ice massage of the web between the thumb and index finger of the hand on the same side as the painful region. Ice massage decreased the intensity of dental pain in the majority of patients. The same authors also found it to be as effective as transcutaneous electric stimulation in reducing lower back pain.^12^ In 2003, Walters *et al*.^10^ investigated the use of ice massage of the large intestine acupressure energy meridian point (LI4) to reduce labor pain during contractions and showed successful reduction in the intensity of labor pains. However, the studies evaluating the effect of cutaneous stimulation on injection and AVF puncture pain have shown conflicting results. Park *et al*.^7^ found that cutaneous stimulation decreased intravenous injection pain in chemotherapy patients with a reduction in both the objective and subjective pain scores. However, they found that in patients on HD, cutaneous stimulation only partly reduced the subjective pain scores and had no effect on the objective pain scores during AVF puncture. The methods used for cutaneous stimulation are, however, not adequately described in these studies, nor are the baseline characteristics well studied in these patients.

The mechanisms involved in the reduction of pain by using cryotherapy can be best explained by the gate control study proposed by Melzac.^8^ There is evidence that cold signals are transmitted to the spinal cord exclusively by A-delta fibers and not by C fibers, which may provide a potential method for differentiating among the multiple feedback systems that mediate analgesia.^12^ Stroking in animal models—massage-like stroking of rats increased withdrawal latencies in response to thermal and mechanical painful stimuli. This effect is in part related to the increased level of oxytocin in the periaquaductal gray (PAG), which can influence the descending antinociceptive system.^15^

In our study, we found that a comparison of day 1 and day 2 pain scores within the control group revealed no significant difference in the objective (*P* = 0.23) or subjective (*P* = 0.89) scores. This proves that there is no significant variation in the pain scores of these two different AV fistula-puncturing procedures in the same individual. It also strengthens the conclusion that the reduction in pain scores within the experimental group was because of the cryotherapy applied during puncturing. It was also found that there was no significant correlation between the AV fistula puncture pain scores and variables such as the duration of AV fistula use and the age of the subjects. We also noted that females reported higher pain scores when compared to males. Jackson *et al*.^16^ studied gender differences in pain perception and found that women typically reported greater sensitivity and less tolerance for experimentally induced noxious stimulation than men.

Our study, however, has a few limitations: i) it is a small sample-sized study and the results need to be confirmed by larger multicentric studies. ii) Also, although patients with diabetic neuropathy were excluded from the study, subjects having diabetes mellitus as a co-morbidity were not uniformly divided between the two randomized groups.

We therefore conclude that cryotherapy is effective in reducing AV fistula puncture pain of hemodialysis patients. AV fistula puncture-related pain intensity was associated with the female sex while no correlation existed with the duration of fistula use (number of punctures undergone), the educational qualification, or the age of the subjects.
